# Deep Learning Deciphers Protein*–*RNA Interaction

**DOI:** 10.1016/j.gpb.2019.11.002

**Published:** 2019-11-23

**Authors:** Ming Li

**Affiliations:** 1 David R. Cheriton School of Computer Science, University of Waterloo, Waterloo, Ontario N2L 3G1, Canada; 2 Ningbo Institute of Information Technology Application, Chinese Academy of Sciences, Ningbo 315000, China

## Background

Protein*–*RNA interaction is ubiquitous in cells and serves as the main mechanism for post-transcriptional regulation. RNA binding proteins (RBPs) not only control which transcripts are translated, but also determine the speed, location, and concentration of mRNA translation, through controlling multiple layers of gene regulation. Base-dominant interaction and backbone-dominant interaction categorize the two main modes of the way RBPs interact with RNA.

There are mainly two approaches to understand protein*–*RNA interaction: experimental techniques and computational methods (Table 1). The former includes high-throughput assays, such as *in vitro* (*e.g.*, RNAcompete) and *in vivo* (*e.g.*, CLIP-HITS) assays, and structural biology approach. However, both technologies have clear limitations: the assay experiments can reveal statistical patterns (*e.g.*, sequence logos) of the binding RNAs to an RBP, but cannot elucidate where and how the RNA interacts with the RBP, whereas the structural biology approach can only capture a snapshot of a specified RNA binding to the RBP, without revealing any statistical property. The computational approach, on the other hand, is still at the early development stage. All the existing machine learning-based methods try to make binary predictions. That is, the state-of-the-art resolution is on predicting if a residue of an RBP is a binding residue or not. Such methods often have high false positive rates, even for known RBPs. For a previously unknown RBP, the predictions from the existing machine learning-based methods and docking-based methods are even less reliable, which hamper their applications in guiding the downstream experimental design.

**Table 1:** Summary of the properties of different experimental and computational techniques for protein*–*RNA interaction studies.

**Property**	**Assays**	**Structural biology**	ML-**based methods**	**Docking-based methods**	**NucleicNet**
Structural information	N	Y	N	Y	Y
Sequence logo	Y	N	Y	N	Y
Binary prediction	N	N	Y	P	Y
RNA-constituent prediction	N	N	N	P	Y
Ability to rank RNAs	Y	P	Y	Y	Y
Ability to identify new RBPs	Y	N	N	P	Y

*Note*: Y, N, and P stand for having, not-having, and partially-having the corresponding property, respectively. ML, machine learning; RBP, RNA-binding protein.

## NucleicNet—RNA-constituent level predictor of protein*–*RNA interaction through deep learning

The new tool, NucleicNet [1], developed by Gao Lab (http://sfb.kaust.edu.sa) at the King Abdullah University of Science and Technology (KAUST), in collaboration with groups in China and USA, is the first-of-its-kind to predict protein*–*RNA interactions at the RNA-constituent resolution. They formulated the problem as a seven-class classification problem, where the label space includes non-site, ribose, phosphate, and four different bases.

For any deep learning approach, data are the most critical component. Lam et al. composed a dataset that contains all the solved protein*–*RNA complex structures in the Protein Data Bank (PDB), and carefully removed the redundant structures and redundant chains, which resulted in a stringent dataset of 175 RNA-binding protein chains. The surface grid points are extracted and the labels are assigned to the grid points by considering the nearest RNA constituents or assigned 0 if the grid point is outside the bound RNA. The FEATURE framework is applied on a contour-manner to extract spatial, physicochemical properties of the local protein surface environment. A 16-level residual network is trained to learn the mapping between the input features and the seven classes. To optimize the problem in a more efficient way, they applied a number of techniques, such as down-sampling of the negative dataset, hierarchical classification, batch normalization, and weight decay.

The authors tested NucleicNet on various tasks, starting from the traditional binary classification, *i.e.*, to predict if a surface residue is an RNA-binding residue or not. Although NucleicNet was trained on the 7-class classification task, when rounding the prediction results to binding/nonbinding, it can still outperform all the sequence-based predictors by a large margin. They then evaluated 7-class classification performance, to which there is no precedent method to compare, through both micro- and macro-performance measures, where ‘micro’ is sample-averaged and ‘macro’ is class-averaged.

**Figure 1:**
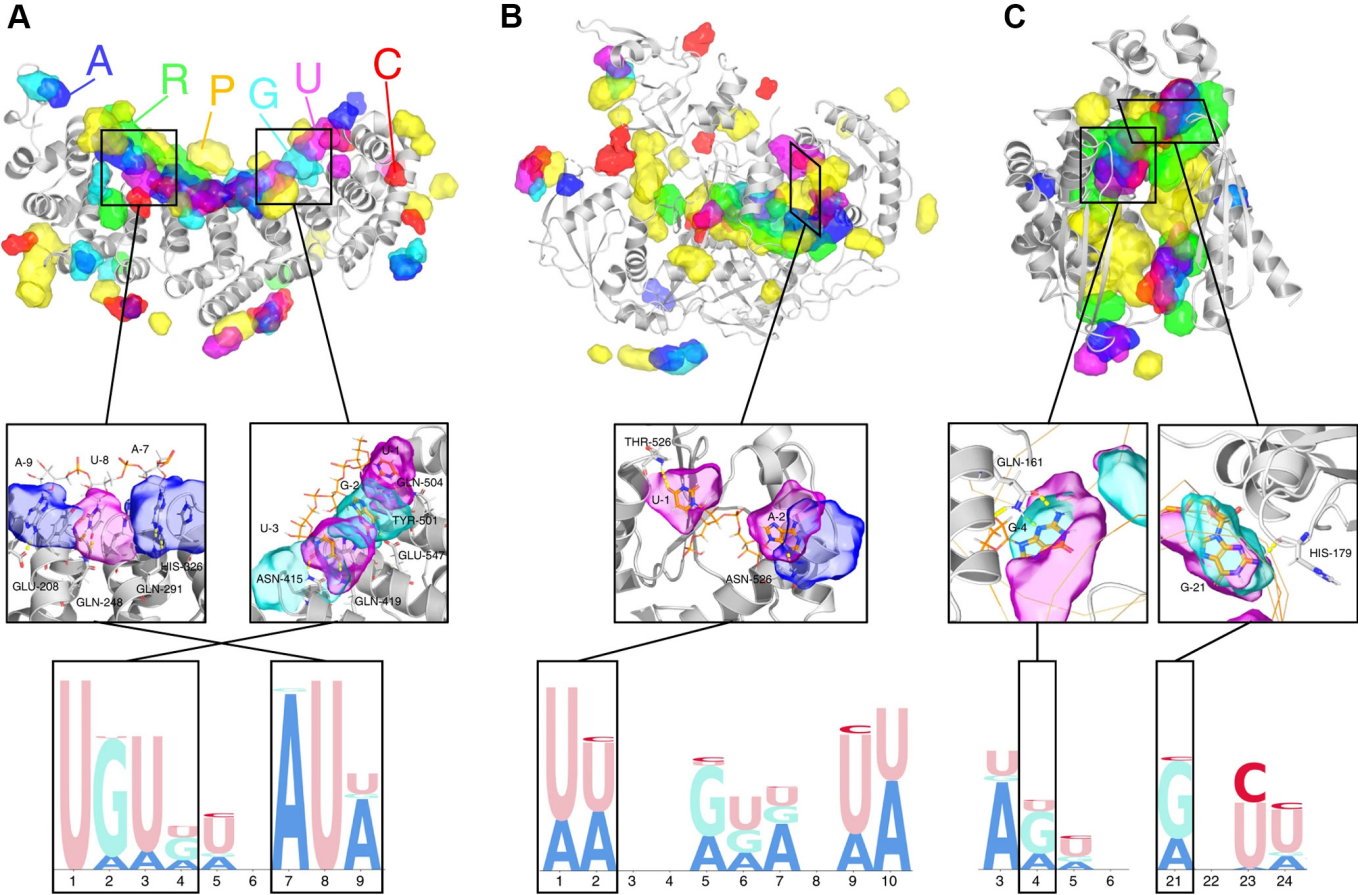
**Three case studies demonstrating the ability of NucleicNet to predict protein*–*RNA interactions** **A.** Fem-3-binding-factor 2 (FBF2), which binds to RNA through base contacts. **B.** Human Argonaute 2 (hAgo2), which binds to RNA through backbone. **C.***Aquifex aeolicus* ribonuclease III (Aa-RNase III), which binds to double-stranded RNA. Upper panel: NucleicNet predictions for query RBPs are shown in the top panels; the detailed views on chemical interactions are shown in the middle panels; and the predicted sequence logo diagrams for the respective query RBPs are shown in the bottom panels. RBP, RNA binding protein. The figure was adopted from [1].

In addition to statistical evaluation, they showed three case studies to demonstrate NucleicNet’s power in revealing complex spatial patterns: Fem-3-binding-factor 2 (FBF2), which binds to RNA through base contacts, human Argonaute 2 (hAgo2), which binds to RNA through backbone, and *Aquifex aeolicus* ribonuclease III (Aa-RNase III), which binds to double-stranded RNA (Figure 1). For FBF2 (Figure 1A), NucleicNet successfully recovered the strong UGUR motif. Interestingly, NucleicNet captures the modest preference for A or U at base 9, which is consistent with recent reports [2], [3], while the complex structure solved in PDB has C at that base. This indicates that NucleicNet can really capture the physicochemical mechanisms in protein*–*RNA interaction through mining from big structural biology data. For both hAgo2 (Figure 1B) and Aa-RNase III (Figure 1C), NucleicNet was able to capture well-known patterns, as well as recently-reported patterns. In all three cases, NucleicNet correctly predicted the binding pockets on the protein surface in an unbiased way, which demonstrates its ability to predict novel RBPs and their binding pockets.

They further validated NucleicNet on *in vitro* and *in vivo* assay data. On the *in vitro* RNACompete datasets, NucleicNet scores on different RNA-binding sequences showed a remarkable level of agreement with the RNACompete position weight matrix scores. NucleicNet was also able to differentiate top scoring sequences from the bottom scoring ones. On the *in vivo* Ago2 immunoprecipitation and siRNA knockdown datasets from different cell lines of both humans and mice, NucleicNet correctly predicted asymmetry in guide strand loading for majority of the cases. These results become even more significant when considering the fact that NucleicNet was never trained on any assay data, and yet it reached a remarkable level of consistency with such high-throughput experiments.

In the case of known RBPs, NucleicNet can be applied to score any given RNA-binding sequence, design the most preferred binding sequence, and draw sequence logos. In the case of proteins with unknown RNA-binding functions, NucleicNet can be applied to check if the protein has a proper RNA-binding site, and if so, what the preferred RNA-binding sequences are. They further provided a webserver for the community to use NucleicNet.

## Discussion

The aforementioned experiments demonstrated the ability of NucleicNet to capture both the statistical and physicochemical properties underlying protein*–*RNA interactions. The deep learning model clearly does much more than ‘memorizing’ the training data. NucleicNet can be potentially applied to understand the binding mechanism and design RNAs for some important RBPs, such as Ago2, m^6^A-responsive RBPs, and RBPs for single guide RNA (sgRNA) in the CRISPR-Cas9 system.

Despite the success of NucleicNet, there are two future directions. First, NucleicNet does not consider the conformational change caused by protein*–*RNA interaction. Thus the input apo structure may undergo a large-scale conformational change to accommodate the binding of the RNA, which would cause the extracted physicochemical features to be imprecise. Second, the idea of NucleicNet can be naturally transferred to model other interactions, such as protein*–*DNA interaction, protein*–*drug interaction, and protein*–*ligand binding. Finally, some ablation studies might help simplify the network.

## Competing interests

The author has declared no competing interests.
